# Factors Influencing Early and Late Mortality in Adults with Invasive Pneumococcal Disease in Calgary, Canada: A Prospective Surveillance Study

**DOI:** 10.1371/journal.pone.0071924

**Published:** 2013-10-08

**Authors:** Leah J. Ricketson, Alberto Nettel-Aguirre, Otto G. Vanderkooi, Kevin B. Laupland, James D. Kellner

**Affiliations:** 1 Department of Pediatrics, University of Calgary, Calgary, Alberta, Canada; 2 Department of Community Health Sciences, University of Calgary, Calgary, Alberta, Canada; 3 Alberta Children’s Hospital Research Institute for Child and Maternal Health, University of Calgary, Calgary, Alberta, Canada; 4 Department of Microbiology, Immunology and Infectious Diseases, University of Calgary, Calgary, Alberta, Canada; 5 Department of Pathology and Laboratory Medicine, University of Calgary, Calgary, Alberta, Canada; 6 Department of Critical Care Medicine, University of Calgary, Calgary, Alberta, Canada; 7 Department of Medicine, University of Calgary, Calgary, Alberta, Canada; Rockefeller University, United States of America

## Abstract

**Background:**

Invasive pneumococcal disease continues to be an important cause of mortality. In Calgary, 60% of deaths occur within 5 days of presenting to hospital. This proportion has not changed since before the era of penicillin. The purpose of this study was to investigate what factors may influence death within 5 days of presentation with pneumococcal disease.

**Methods and Findings:**

Demographic and clinical data from the CASPER (Calgary Area Streptococcus pneumoniae Epidemiology Research) study on 1065 episodes of invasive pneumococcal disease in adults (≥18 years) from 2000 to 2010 were analyzed. Adjusted multinomial regression was performed to analyze 3 outcomes: early mortality (<5 days post-presentation), late mortality (5-30 days post-presentation), and survival, generating relative risk ratios (RRR). Patients with severe disease had increased risk of early and late death. In multinomial regression with survivors as baseline, the risk of early death increased in those with a Charlson index ≥2 (RRR: 6.3, 95% CI: 1.8-21.9); the risk of late death increased in those with less severe disease and a Charlson ≥2 (RRR: 6.1, 95% CI: 1.4-27.7). Patients who never received appropriate antibiotics had 5.6X (95% CI: 2.4-13.1) the risk of early death. Risk of both early and late death increased by a RRR of 1.3 (95% CI: 1.2-1.4) per 5-year increase in age. In multinomial regression, there were no significant differences in the effects of the factors tested between early and late mortality.

**Conclusions:**

Presenting with severe invasive pneumococcal disease, multiple comorbidities, and older age increases the risk of both early and late death. Patients who died early often presented too late for effective antibiotic therapy, highlighting the need for an effective vaccine.

## Introduction

*Streptococcus pneumoniae* is an important pathogen in both the developed and developing world, especially in the very young and very old. Invasive pneumococcal disease (IPD) continues to cause death in Canada, primarily among the elderly. Evidence is inconsistent regarding the effectiveness of the current 23-valent polysaccharide vaccine for pneumococcal disease in adults, especially in high-risk adults [1-5].

In 1964, Austrian and Gold showed that 60% of deaths in patients with IPD occurred within 5 days of presentation, regardless of treatment with penicillin [6]. In Calgary between 2000 and 2009, 60% of deaths occurred within 5 days of presenting to a healthcare center (unpublished data). Thus, the proportion of deaths that occur early after presentation with IPD does not appear to have changed over nearly five decades. It was hypothesized that those patients who die early present with more severe disease, causing them to be less responsive to treatment and to deteriorate more quickly. The purpose of this study was to examine the differences between early mortality, late mortality, and survival in order to better understand the progression of IPD.

Previous studies that have examined early mortality have looked at all-cause community-acquired pneumonia (CAP) or bacteremia [7-10]. Marrie et al. previously evaluated mortality in IPD across the province of Alberta (including some cases from the present study); however, the analysis was not as comprehensive, fewer years were reported (2000 to 2004), and early and late mortality were examined in separate logistic regression models rather than the same model concurrently [11]. Therefore, the current study provides a unique perspective on IPD mortality.

## Methods

### Surveillance

The Calgary Area Streptococcus pneumoniae Epidemiology Research (CASPER) team has been collecting data on all episodes of IPD in Calgary and area since 1998. When a culture positive for *S. pneumoniae* is found, Calgary Laboratory Services (which serves the entire region) notifies the research team and the patient is enrolled. A standard case report form is used to collect data from the patient’s chart, and an interview is conducted with the patient or surrogate. Patients who cannot be contacted for consent have only a chart review done. The only patients who are excluded are those who refuse consent, those with non-invasive infections, and those who present to a Calgary hospital but live outside of the Calgary Zone of Alberta Health Services.

This study involved adults ≥ 18 years of age from the CASPER database who presented between January 1, 2000 and December 31, 2010. For the current study, if more than one invasive *S. pneumoniae* isolate was obtained from a patient within a single episode, only the first isolate was considered. If a non-blood invasive isolate was collected, then the non-blood isolate was considered over the blood isolate. If a patient had a second positive culture more than 30 days after the first, these were considered to be separate episodes of IPD, and both were included. In all, there were 38 second or subsequent episodes that occurred in 33 patients.

### Ethics Approval

CASPER has ethics approval from the Conjoint Health Research Ethics Board (CHREB) of the University of Calgary and Calgary Zone of Alberta Health Services for data collection and analysis. Written, informed consent was obtained from all participants or their next of kin (for those who died) and the original signed consent forms have been retained and stored according to the policies of the CHREB. For the small number of patients who could not be contacted or refused consent, information was obtained from the "Notifiable Disease Report Form," which is a form mandated to be completed by the Province of Alberta Department of Health on all notifiable diseases, including all cases of invasive *S. pneumoniae*. The CHREB approved all of these procedures.

### Laboratory Analysis

*Streptococcus pneumoniae* is isolated by the Calgary Laboratory Services and identified using colony morphology, alpha hemolysis, optochin susceptibility, and bile solubility. All viable isolates were serotyped and had antibiotic susceptibilities conducted. The Provincial Laboratory for Public Health (Edmonton, Alberta) serotyped the isolates using the Quellung reaction as part of routine testing with serotype specific anti-sera from Statens Serum Institute (Copenhagen, Denmark). Minimum inhibitory concentrations (MICs) were determined for all isolates using broth microdilution following the Clinical Laboratory Standards Institute (CLSI) guidelines for susceptibility [12]. The isolates were classified as susceptible or non-susceptible for the purpose of analysis. Intermediate and resistant isolates were considered to be non-susceptible. For antibiotics not measured in the MicroScan MICroSTREP plus® panel (Siemens, USA) the MICs were inferred, where appropriate, from other antibiotic MICs [12].

### Data Analysis

Univariable analysis was conducted using Z test for difference in proportions for categorical variables and t-test for continuous variables to compare early mortality to late mortality groups, and overall mortality to survival with regards to several potential risk factors that were chosen *a priori* based on the current literature on risk factors for mortality and IPD. Age (continuous), gender, primary diagnosis (meningitis vs. non-meningitis), Charlson comorbidity index (0 vs. ≥1), and current smoking status were analyzed. The Charlson comorbidity index was calculated with the original method that excludes age [13]. This allowed us to examine the independent effects of age.

Multinomial logistic regression was used to analyze the 3 outcomes—early mortality (<5 days post-presentation), late mortality (5-30 days post-presentation), and survival—in a single model. This method allowed for less loss of information and a more uniform approach to analysis of 3 outcomes. In multinomial regression each outcome is modeled relative to the baseline outcome group: survival. Therefore, relative risk ratios (RRR) are reported rather then odds ratios or risk ratios.

**Figure 1:**
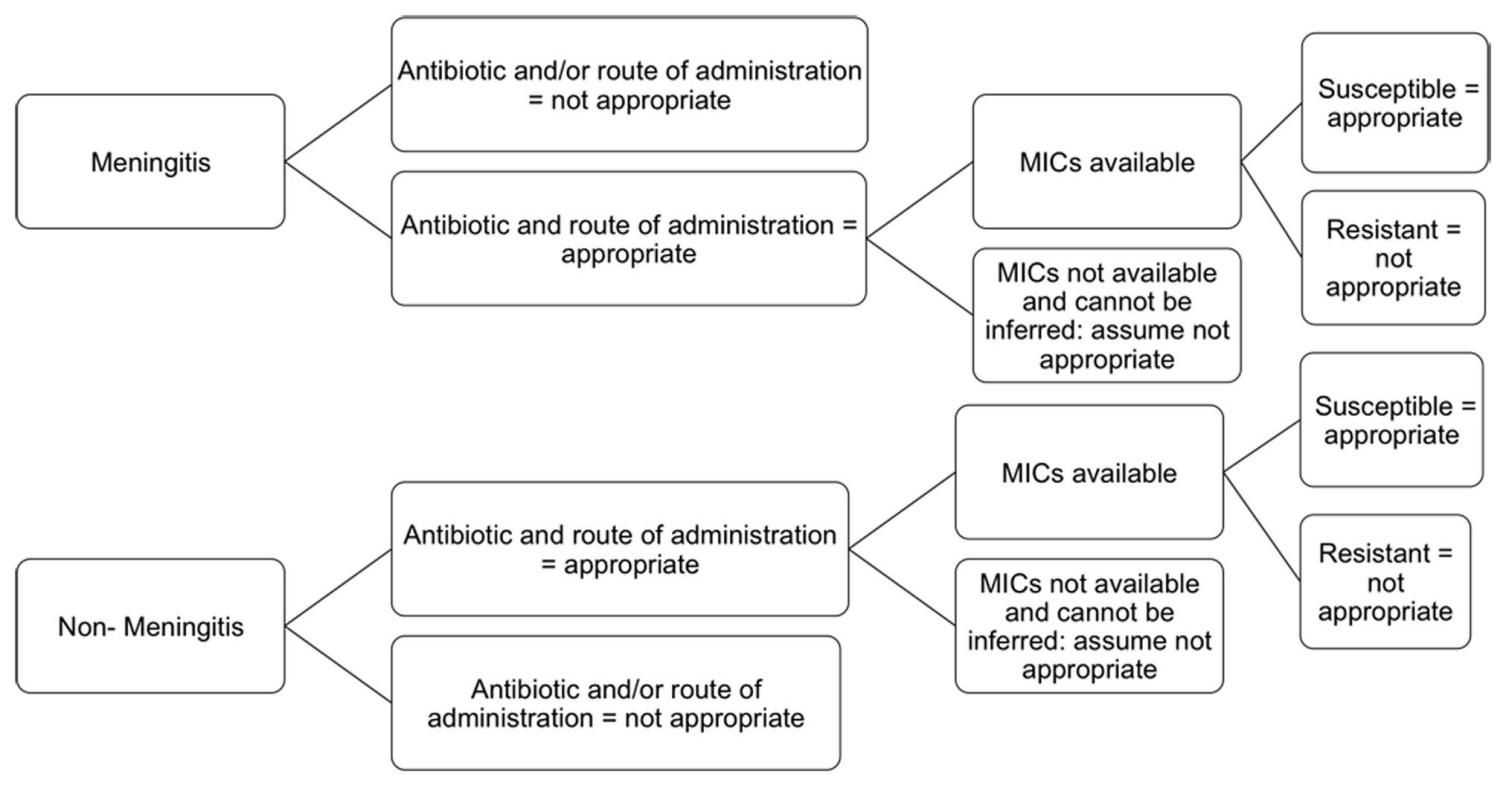
Classification of Antibiotic Appropriateness Accounting for Isolate Susceptibilities.

The primary independent (predictor) variable was ICU admission and/or mechanical ventilation, which were chosen as proxies for disease severity. The models were adjusted for six covariates including age (continuous), gender, primary diagnosis (meningitis, pneumonia/empyema, bacteremia/other invasive), smoking status (current vs. former/never/missing), Charlson comorbidity index (0, 1 or ≥2) [13], and time to first dose of appropriate antibiotic therapy. Antibiotics were considered appropriate if they were an appropriate treatment option for *S. pneumoniae* based on previous literature and the expert opinion of 3 physicians (JK, OV, KL), and if the infecting strain was susceptible to that specific antibiotic (Figure 1). The times that represented the shortest period from presentation to appropriate treatment for each patient were then grouped into < 24 hours to treatment, 24-48 hours, > 48 hours, and no appropriate treatment received.

We included biologically plausible pair-wise interaction terms in the model to allow for evaluation of effect modification. In the informed model interaction terms were included for all of the six covariates as effect modifiers of severity, as well as interaction terms for possible joint confounding from age and primary diagnosis, Charlson index and time to appropriate treatment, primary diagnosis and time to appropriate treatment, and age and time to appropriate treatment. These “joint confounding” terms also allow for investigation of whether these covariates may modify each other as independent risk factors. The final models were achieved through backward elimination. Effect modification was assessed based on significance of interaction terms.

The multinomial analysis was run both with and without serotype 5 outbreak cases in order to ensure the results were not skewed by the serotype 5 outbreak that occurred in 2006-2007 [14]. Analysis was conducted using STATA Intercooled statistical software, version 11.

## Results

### Population

**Figure 2:**
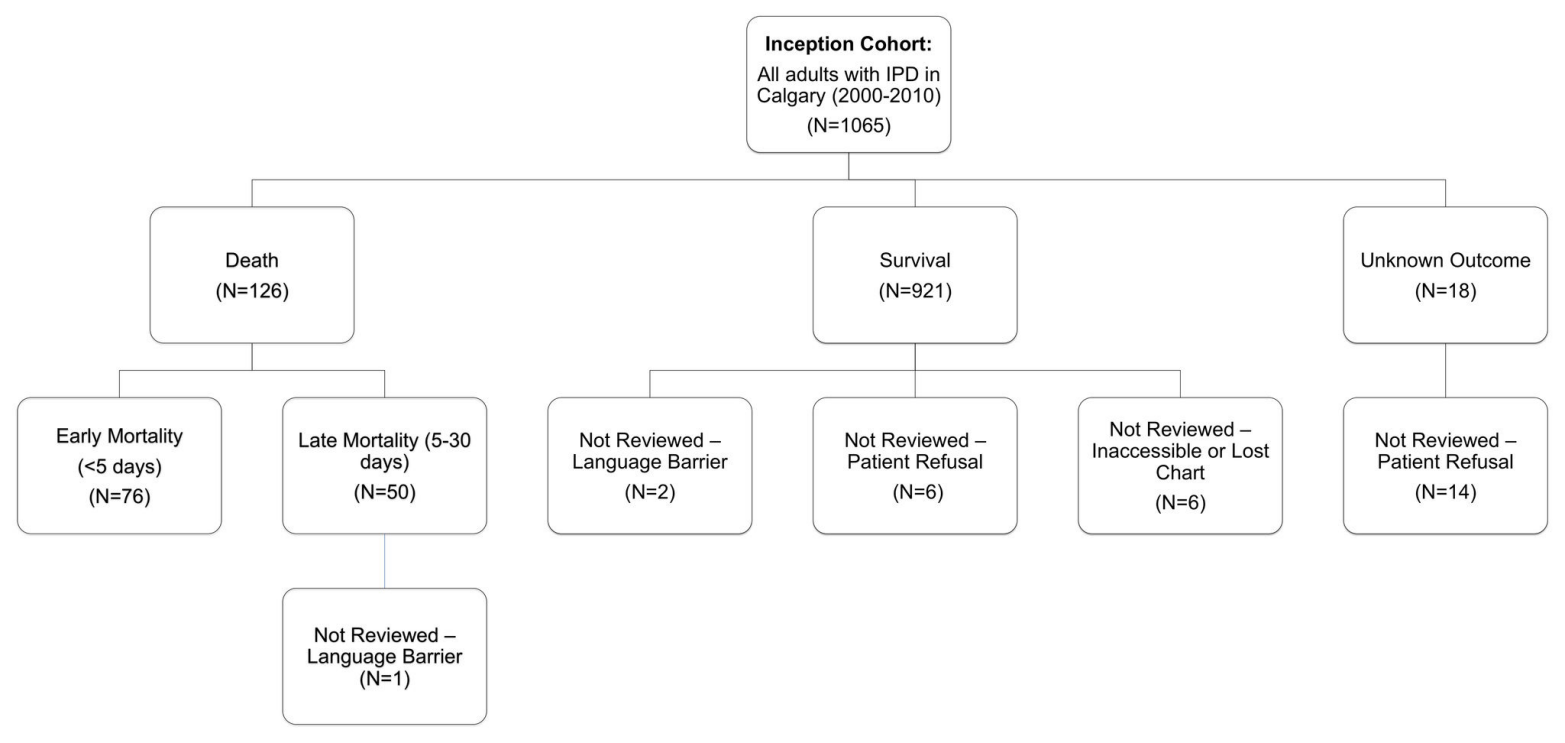
Outcomes for Eligible Cohort of Calgary Adults with IPD from 2000-2010.

There were a total of 1065 episodes of IPD in 1026 Calgary adults from 2000 to 2010 (Figure 2). Of 1065 episodes, 1034 (97.1%) had complete information and were included in the multivariable analysis. Twenty-nine eligible people did not have full reviews done; (Table 1). Twenty patients refused participation in the study and 3 reviews were not done due to language barriers with the patient and next of kin that precluded consent, but outcome data was available. Six charts were not available for review and two charts were missing some information required for the multivariable analysis. All patients had basic information from Notifiable Disease Reports (made to the provincial public health department) and lab reports and were included in descriptive analyses where information was available. There were no significant differences in terms of age group (< 65 or ≥ 65) or gender between those excluded due to missing chart reviews or refusal and those included in the study.

**Table 1:** Population Characteristics.

**Characteristic**	**Characteristic sub-category**	**N (% of total)**
Overall population N		1065
Population with full reviews		1034 (97.0)
Age group (N=1065)	18-64 years	767 (72.0)
	65-84 years	244 (22.9)
	85+ years	54 (5.1)
Gender (N=1065)	Male	612 (57.5)
	Female	453 (42.5)
Comorbidities (N=1045)	Charlson comorbidity index=0	393 (37.6)
	Charlson comorbidity index=1	278 (26.6)
	Charlson comorbidity index ≥2	374 (35.8)

The mean age of the sample was 54.8 years (SD 17.7). Of these, 42.5% were female and 57.5% were male. Of 1,058 patients with a recorded outcome, 126 died within 30 days of presentation giving a case-fatality rate of 11.9%. Of those who died, 60.3% died within 5 days of presentation with IPD, which represented the cut-off for early mortality. Overall, 37.6% had a Charlson index of 0, 26.6% had a Charlson index of 1 and 35.8% had a Charlson index of 2 or more. Among those with a Charlson index of 2 or higher, 60.4% were under the age of 65 years. The largest proportion of episodes (74.9%) had pneumonia as the primary diagnosis. Meningitis accounted for 4.1% of the diagnoses.

Despite changes in serotype distribution since the introduction of the vaccine, there was no significant change in case fatality rates among adults. In 2000/2001 (pre-vaccine) the case fatality rate was 13.7% compared to 11.4% in 2003-2010 (post-vaccine) (P-value=0.3998).

### Serotypes

Of the 1053 cases for which serotypes were available, 266 (25.3%) were PCV7 serotypes (4 (7.1%), 6B (3.0%), 9V (2.8%), 14 (5.4%), 18C (2.3%), 19F (1.9%) and 23F (2.8%)). Additionally, 639 (60.7%) were PPV23 serotypes not also in PCV7 (1 (1.2%), 3 (7.8%), 5 (16.6%), 7F (3.6%), 19A (3.4%), 8 (8.1%), 9N (3.6%), 10A (0.6%), 11A (2.4%), 12F (3.4%), 15B (0.6%), 17F (0.9%), 20 (0.4%), 22F (6.6%), 33F (1.5%). There were also 148 (14.1%) cases caused by serotypes not in any PCV7 or PPV23 including 6A (3.1%), 7C (0.2%), 9L (0.4%), 10F (0.2%), 11B (0.5%), 11C (0.1%), 11F (0.1%), 12A (0.1%), 13 (0.1%), 15A (0.6%), 15C (0.4%), 16F (1.3%), 18B (0.1%), 21 (0.1%), 22A (0.1%), 23A (0.6%), 23B (0.2%), 28A (0.2%), 29 (0.1%), 31 (1.5%), 33A (0.4%), 34 (0.8%), 35A (0.1%), 35B (1.1%), 35C (0.1%), 35F (0.6%), 38 (0.8%), not typeable (0.4%). The most prevalent serotypes causing disease in this sample were serotype 5, 8, 3, 4, 22F, 14, 7F, 9N, 19A, and 12F. Of the 126 deaths, the largest proportion were caused by serotype 3 (12.7%) and 22F (9.5%). Serotype information was not included in the multinomial models.

### Risk Factors

In the univariable analysis comparing mortality to survival groups, significant risk factors for death (at significance level alpha=0.05) included ICU admission (26% vs. 7%), mechanical ventilation (32% vs 7%), female gender (16% vs. 9%), Charlson comorbidity index greater than 1 compared to Charlson index of 0 (15% vs. 7%), and meningitis diagnosis compared to any other diagnosis (40% vs 11%). Age was also a significant risk factor for mortality with a mean age of 53.3 (SD: 17.3) for survivors and 65.6 (SD: 16.8) for those who died. There were no significant differences between early and late mortality in the univariable analysis.

The multinomial analysis was performed using 1034 of 1065 episodes who had complete information for all covariates (97.1%). Tables 2-4 show risk factors associated with early mortality relative to survival, late mortality relative to survival, and early mortality relative to late mortality in patients with IPD.

**Table 2:** Risk Factors Associated with Early Mortality Compared to Survival in Patients with IPD in Calgary.

**Risk Factor**	**Risk Factor Sub-category**	**RRR of Early Mortality to Survival (95% CI)^a^**	**P-value**
**Disease Severity**	Less Severe Disease	Reference group	
	More Severe Disease	13.7 (3.3-56.9)	<0.001
**Comorbidities**	Charlson comorbidity index=0	Reference group	
	Charlson comorbidity index=1	2.6 (0.7-10.4)	0.164
	Charlson comorbidity index≥2	6.3 (1.8-21.9)	0.004
**Age**	Per 5 year increase	1.3 (1.2-1.4)	<0.001
**Gender**	Female	Reference Group	
	Male	0.6 (0.3-0.9)	0.03
**Primary Diagnosis**	Bacteremia/Other Invasive	Reference group	
	Pneumonia/Empyema	0.5 (0.3-1.2)	0.115
	Meningitis	1.7 (0.5-5.4)	0.369
**Time to receipt of appropriate antibiotic treatment**	<24 hours post-presentation	Reference group	
	24-48 hours post-presentation	1.0 (0.3-3.2)	0.948
	>48 hours post-presentation	-^b^	-^a^
	No receipt of appropriate antibiotics	5.6 (2.4-13.1)	<0.001

a RRR=Relative Risk Ratio 95% CI = 95% Confidence Interval; b. No inferences can be made with this estimate due to a zero cell.

**Table 3:** Risk Factors Associated with Late Mortality Compared to Survival in Patients with IPD in Calgary.

**Risk Factor**	**Risk Factor Sub-category**	**RRR Late Mortality to Survival (95% CI)^a^**	**P-value**
**Disease Severity Modified by Comorbidities**	Less severe disease and Charlson comorbidity index =0	Reference group	
	More severe disease and Charlson comorbidity index=0	21.1 (4.2-105.7)	<0.001
	Less severe disease and Charlson comorbidity index=1	-^b^	-^a^
	More severe disease and Charlson comorbidity index =1	22.6 (4.5-113.9)	<0.001
	Less severe disease and Charlson comorbidity index ≥2	6.1 (1.4-27.7)	0.018
	More severe disease and Charlson comorbidity index ≥2	22.4 (4.8-103.9)	<0.001
**Age**	Per 5 year increase	1.3 (1.1-1.4)	<0.001
**Gender**	Female	Reference group	
	Male	0.6 (0.3-1.2)	0.163
**Primary Diagnosis**	Bacteremia/other invasive diagnosis	Reference group	
	Pneumonia/empyema diagnosis	1.0 (0.3-3.1)	0.990
	Meningitis	3.3 (0.8-14.1)	0.099
**Time to receipt of appropriate antibiotic treatment**	<24 hours post-presentation	Reference group	
	24-48 hours post-presentation	1.2 (0.3-4.3)	0.776
	>48 hours post-presentation	1.6 (0.4-6.1)	0.463
	No receipt of appropriate antibiotics	0.8 (0.1-6.7)	0.863

a RRR=Relative Risk Ratio 95% CI = 95% Confidence Interval; b. No inferences can be made with this estimate due to a zero cell.

**Table 4:** Risk Factors Associated with Early Mortality Compared to Late Mortality in Patients with IPD in Calgary.

**Risk Factor**	**Risk Factor Sub-category**	**RRR Early Mortality to Late Mortality (95% CI)^a^**	**P-value**
**Disease Severity**	Less Severe Disease	Reference group	
	More Severe Disease	0.7 (0.1-5.2)	0.685
**Comorbidities**	Charlson comorbidity index=0	Reference group	
	Charlson comorbidity index=1	-^b^	-^a^
	Charlson comorbidity index≥2	1.0 (0.1-7.0)	0.983
**5 year increase in Age**		1.0 (0.9-1.2)	0.745
**Gender**	Female	Reference group	
	Male	0.9 (0.4-1.8)	0.724
**Primary Diagnosis**	Bacteremia/other invasive diagnosis	Reference group	
	Pneumonia/empyema diagnosis	0.5 (0.2-1.9)	0.339
	Meningitis	0.5 (0.1-2.6)	0.413
**Time to receipt of appropriate antibiotic treatment**	<24 hours post-presentation	Reference group	
	24-48 hours post-presentation	0.9 (0.2-4.2)	0.854
	>48 hours post-presentation	-^b^	-^a^
	No receipt of appropriate antibiotics	6.7 (0.8-56.1)	0.080

a RRR=Relative Risk Ratio 95% CI = 95% Confidence Interval; b. No inferences can be made with this estimate due to a zero cell.

Patients with more severe IPD had increased risk of early death and females were at increased risk of early death compared to males. Patients who never received appropriate antibiotics had increased risk of early death. We investigated these 12 cases individually and found that 3 died in the ER within 5 hours of presenting before antibiotics were provided, 2 appeared to be designated as palliative on presentation and may not have received antibiotics for this reason, 2 were meningitis cases that never presented to hospital, 2 had antibiotics ordered, but never given, and 3 were given antibiotics that were not appropriate for the infection. None of the cases involved treatment failure due to antibiotic resistant bacteria, but most had other comorbidities – including cancer, illegal drug use, and myocardial infarction – contributing to the outcome.

The relationship between late mortality and disease severity was modified by a Charlson comorbidity index ≥ 2; therefore, separate estimates of the RRR for disease severity are reported for each level of effect modifier (Table 3).

No one who died late had a Charlson index of 1 and less severe disease (Table 3). Therefore, a RRR for late death cannot be estimated for this group. Similarly, no one who died early received antibiotics greater than 48 hours after presentation (Table 2). Again, no estimate of effect can be made from this RRR. These same two estimates cannot be considered in early compared to late mortality for the same reasons (Table 4).

Increasing age was a risk factor for both early and late mortality. For every 5 year increase in age the relative risk of both early and late mortality increases by 1.3 times (95% CI: 1.2-1.4).

When the multinomial logistic regression was run without the serotype 5 outbreak cases the same factors were significant and the results did not change except for female gender was no longer a significant risk factor for early mortality.

## Discussion

Although the case-fatality rate of IPD has declined considerably, the proportion of early deaths is unchanged over five decades [6]. We found some differences between the patterns of early and late deaths, compared with survival. Early death was more likely with severe disease, higher comorbidity (Charlson index ≥ 2), increased patient age, female gender, and if appropriate antibiotics were never received. Late death was more likely with more severe disease regardless of level of comorbidity and increased patient age. When early and late deaths were compared, never receiving appropriate antibiotics was a near significant factor for early deaths but no other significant differences were found.

There was a broad distribution of serotypes causing IPD in this study. The most common serotypes causing disease in Calgary adults were serotypes 5 and 8. This is different from normal patterns, as there was an outbreak of serotype 5 among homeless adults in 2006-2007 and an outbreak of serotype 8 in 2005 [14]. However, when run without the outbreak cases our results and conclusions did not change.

Serotype was not considered in the multivariable analysis for this study. This study focused on patient factors. Knowledge of the infecting serotype does not change the clinical management of IPD, while some host factors may be controlled to increase a patient’s chance of survival. Most of the research indicating an increased risk of death associated with serotypes is comparing specific serotypes to a reference serotype [15,16]. Inevitably when comparing a serotype with a high tendency to cause death against a serotype with a low tendency to cause death there will be an association. Invasive pneumococcal disease is caused by a diversity of serotypes and it is unlikely that single serotypes would have a large confounding effect on the current study results. When serotypes were analyzed as a group, Alanee et al. found host factors to be more associated with death than serotype groups [17]. Also, antimicrobial resistance was considered for each strain to determine antimicrobial appropriateness.

Risk factors for early mortality relative to survival in the multinomial analysis included presentation with severe disease, high Charlson comorbidity index, increasing age, and never receiving appropriate antibiotics for IPD. In the current study, females have increased risk of early death. One study by Vallès et al. in 2006 also found increased risk of death among females relative to males hospitalized with pneumonia due to *S. pneumoniae* (OR: 9.1, 95% CI 1.3-61.2) [18]. This study found that women seemed to be admitted to hospital less often, but those who are admitted present with more severe pneumonia. Several previous studies have shown male sex to be a risk factor for IPD [19,20], but only one paper was found showing male sex to be a risk factor for death due to IPD [15]. Further research is needed to understand what differences may cause women to present less often with IPD, but with increased risk of death.

The relationship between disease severity and late death was modified by Charlson index. In patients with less severe disease a Charlson index of ≥ 2 was a risk factor for late mortality. Severe disease increased risk of late death regardless of Charlson index, with similar RRR for all levels of Charlson index and severity. There were no statistically significant differences between the RRR for early and late death in the multinomial logistic regression.

Increasing age was a risk factor for both early and late mortality; however, the risk increase with age was less pronounced than the increase in risk due to comorbidities and presentation with more severe disease. For example, according to the current results, an 80-year-old has 3.5 times the risk of mortality compared to an 18-year-old when considering all other factors such as comorbid conditions and disease severity. A recent study by Barnett et al. showed that although the presence of multiple comorbidities increases with age, over half the people with multiple comorbidities are under 65 years of age and our study found the same [21].

We also found a significant association between early mortality and never receiving appropriate antibiotics. None of these were due to treatment failure with antibiotic resistant bacteria; therefore, these results indicate a need for a more effective vaccination for adults. Studies of the 23-valent pneumococcal polysaccharide vaccine (PPV-23) suggest that it is not effective, especially among high-risk adults with comorbid conditions [5,22,23]. The role and potential impact of the 13-valent pneumococcal conjugate vaccine, which has recently been licensed for use in adults aged 50 years and older in Canada and other countries, remains to be determined, but our results support the use of a vaccine in adults, particularly those with underlying comorbid conditions, as they are at increased risk for both early and late mortality.

### Strengths and Limitations

Through the use of multinomial analysis, risk factors involved in early mortality and late mortality were captured simultaneously in a single model, which is a unique analysis to the current IPD literature. Because IPD is a relatively rare disease in Canada, having eleven years worth of data allowed for a larger sample size and the ability to adjust for a greater number of variables in the multinomial model. Effect modification was explored, which is not often considered in the current literature on IPD mortality.

Early and late mortality are measured from the time of patient presentation at a hospital. The true measure of early and late mortality from the time of infection is difficult to determine. However, this is the reason for including disease severity as the primary exposure for the analysis, as it was hypothesized *a priori* that late presentation with severe disease may be the reason for early mortality. Furthermore, time of presentation is a very clinically relevant time point. Disease severity was an important factor in risk of both early and late mortality, and there was no difference in severity between the two. This suggests that progression of symptoms prior to presentation may be the most important factor influencing early or late death. Patients who die early may have more progressive, and perhaps longer duration of symptoms prior to presentation. Our study was limited in that there was no accurate way to measure duration of symptoms prior to presentation, which may have resulted in lead time bias.

There are limitations to using ICU admission and mechanical ventilation as proxies for disease severity. Some elderly patients may have a care designation that causes them to not be admitted to the ICU despite having severe disease and death. If this misclassification occurred the bias would likely be differential and would result in a decreased association between disease severity and early or late mortality. Therefore, this could cause an underestimation of the true effect.

It was not possible to compare mono versus dual antibiotic therapy when considering the time to treatment. The literature is controversial as to whether dual therapy is a more effective method of treatment for preventing death due to IPD [24].

Vaccination status could not be examined as a covariate, because it is based on patient recall and is missing for many patients. The PPV-23 vaccine is controversial with regards to its effectiveness, particularly among people with comorbidities; therefore, it may not be a relevant factor. The PPV-23 is also not consistently given in Calgary. The most likely people to have received the vaccination are those who had a similar infection previously. According to an Alberta study, the PPV-23 vaccine is ineffective at preventing long term morbidity and mortality in patients who have previously been infected with *S. pneumoniae* [5].

Several studies involving sepsis and pneumonia have shown that most deaths occur early [6-8,10,25]. The initial hypothesis for this study was that patients who die early present later in the course of the disease and with a more severe clinical state. This study showed early mortality was highly associated with severe disease. However, late death was also associated with severe disease. Length of symptoms before presentation could not be evaluated to determine whether more severe disease at presentation correlated with longer duration of symptoms prior to presentation. However, these results highlight the importance of preventive action such as vaccination in order to reduce both early and late mortality. This also raises the issue of developing an effective pneumococcal vaccine that targets the most important strains relevant to adults. While PCV13 has been licensed for use in adults, the use of the vaccine is unregulated, so uptake is likely to be poor until a public health intervention is provided that ensures regular vaccination of high risk adults with comorbidities.

In conclusion, while age is an important risk factor for mortality due to IPD with the RRR increasing with increasing age, the largest risk factors influencing both early and late death due to IPD appear to be presentation with severe disease and the presence of comorbid conditions.
